# Efficacy and safety of orlistat in male patients with overweight/obesity and hyperuricemia: results of a randomized, double-blind, placebo-controlled trial

**DOI:** 10.1186/s12944-024-02047-7

**Published:** 2024-03-11

**Authors:** Shuang Liu, Xiaojing Lin, Minghao Tao, Qi Chen, Hang Sun, Yali Han, Shaoling Yang, Yining Gao, Shen Qu, Haibing Chen

**Affiliations:** 1 Department of Endocrinology and Metabolism, Shanghai Tenth People’s Hospital, Tongji University School of Medicine, 301 Middle Yanchang Road, Shanghai, 200072 China; 2 Shanghai Center for Thyroid Disease, Shanghai Tenth People’s Hospital, Shanghai, 200072 China; 3 Department of Endocrinology and Metabolism, Shanghai Sixth People’s Hospital, Shanghai Jiao Tong University School of Medicine, 227 Chongqing South Road, Shanghai, 200025 China

**Keywords:** Hyperuricemia, Orlistat, Uric acid, Gout flare

## Abstract

**Background:**

Obesity is associated with elevated serum uric acid (SUA) levels and frequent gout flares. Losing weight can reduce the SUA level and gout flares. The effect of orlistat on SUA levels and gout flares in patients with overweight/obesity and hyperuricemia (HUA) has not been extensively studied. This study investigated the effects of orlistat on SUA levels and gout flares compared to placebo in overweight and obese patients with HUA.

**Methods:**

A total of 72 Chinese patients with overweight/obesity and HUA were randomly divided into a placebo group (35, 48.6%) and an orlistat group (37, 51.4%); the trial lasted 12 weeks. The primary endpoints were the relative changes in body weight, the SUA level, and gout flares in the per-protocol population.

**Results:**

Orlistat reduced the proportion of patients with gout flares (log-rank *P* = 0.023, hazard ratio = 0.31, 95% confidence interval 0.11–0.85). There was no significant difference in SUA level between the two groups. The average weight loss of the orlistat group was 2.85 kg, and the average weight loss of the placebo group was 0.76 kg. The weight loss in the orlistat group was significantly greater than that in the control group (*P* < 0.05).

**Conclusions:**

This study is the first to demonstrate that orlistat has no significant effect on SUA levels in patients with overweight/obesity and HUA. The utility of orlistat as an adjunct therapy to prevent gout flares during weight loss in patients with HUA was emphasized.

**Trial registration:**

Clinicaltrials.gov NCT05496075.

**Supplementary Information:**

The online version contains supplementary material available at 10.1186/s12944-024-02047-7.

## Introduction

Hyperuricemia (HUA) is a metabolic disease caused by abnormal purine metabolism and/or reduced excretion of uric acid from the body. The prevalence of HUA in China is currently 13.3% and is increasing in younger people. As society and lifestyles change, the prevalence of HUA will further increase [1]. Excess serum uric acid (SUA) may be deposited in articular and non-articular structures to form monosodium uric acid crystals, leading to gout flares [2] manifested by sudden onset of joint pain, erythema, fever, swelling, and dysfunction [3]. In addition, HUA is closely associated with metabolic syndrome, type 2 diabetes, hypertension, cardiovascular disease, and chronic kidney disease and is an independent risk factor for premature mortality [4–6].

HUA is considered to be a multifactorial chronic disease influenced by genetic, environmental and metabolic factors [7]. In particular, excess body weight is a major risk factor for HUA. Epidemiological studies have shown that for every 4-unit increase in body mass index (BMI), the SUA level rises by 250 µmol/L, and the risk of HUA increases 7.49-fold [8]. An animal study found that obesity promoted increased uric acid production via an effect of hypoxia on adipocytes [9]. The long-term effects of weight loss include a lower uric acid level and relief from gout flares [10]. Given these findings, weight loss is warranted for patients with overweight/obesity and HUA.

Orlistat is a potent and irreversible inhibitor of lipase that blocks the absorption of triglycerides from the diet [11]. Orlistat has been approved by the China National Medical Products Administration for the treatment of obese patients. In clinical trials, orlistat has been shown to be effective in terms of improving obesity and related diseases such as metabolic fatty liver disease [12, 13]. However, the effect of orlistat on SUA levels in obese patients remains controversial. In obese patients with metabolic fatty liver disease, there was no significant difference in the change in SUA between orlistat and control groups [13, 14]. Orlistat combined with Diane-35 significantly reduced SUA levels in overweight and obese patients with polycystic ovary syndrome (PCOS), but there was no significant difference compared with Diane-35 treatment alone [15]. Orlistat combined with a low-calorie diet (LCD) significantly improved SUA levels in patients with type 2 diabetes compared to LCD alone [16]. In summary, there is no conclusive evidence for any effect of orlistat on SUA levels. Studies on the effects of orlistat on SUA levels and gout flares in subjects with overweight/obesity and HUA have not been reported; further research is needed.

The primary objective of this study was to prospectively evaluate the ability of orlistat to lower urate levels and reduce gout flares in patients with overweight/obesity and HUA; we sought to inform clinicians about treatment choices.

## Patients and methods

### Study design

This randomized, double-blind, placebo-controlled trial was designed to compare the efficacy of orlistat and placebo in Chinese patients with overweight/obesity and HUA. Both orlistat and placebo were provided by Hangzhou Zhongmei-Huadong Pharmaceutical Co. Ltd. (China). From August 2022 to December 2023, a total of 72 subjects with overweight/obesity and HUA were recruited in the Endocrinology and Metabolism Department of the Shanghai Tenth People’s Hospital, of whom 55 (76.39%) were gout patients. The primary endpoints were the relative changes in SUA levels and gout flares from baseline to week 12 in a per-protocol population. A gout flare is defined as patient-reported pain that requires rescue medication based on joint symptoms and the nature of the flare. Flares were recorded in patient diaries and by the investigator during follow-up. Secondary endpoints included changes in body weight; waist circumference; body fat composition; glucose metabolism; levels of liver enzymes, insulin, C-peptides, and lipid biomarkers; liver fat content; and the degree of liver fibrosis from baseline to week 12 in the per-protocol population. All study data were systematically collected by the investigator using a custom applet.

Each participant signed an informed consent form prior to entering the study, which was conducted in accordance with the Declaration of Helsinki and the Guidelines for Good Clinical Practice. The study was approved by the Clinical Research Ethics Committee of the Tenth People’s Hospital affiliated with Tongji University. The study has been registered at ClinicalTrials.gov with the identification code NCT 05496075.

### Eligibility

The inclusion criteria were: aged 18–65 years; male patient with HUA; and, BMI (weight in kilograms divided by the square of the height in meters) ≥ 25.0 kg/m^2^. The definitions of gout and HUA were based on the American College of Rheumatology/European Alliance of Associations for Rheumatology gout classification criteria [17].

The exclusion criteria were: gout flares in the last 2 weeks; abnormal liver function, i.e., alanine aminotransferase (ALT) or aspartate aminotransferase (AST) levels ≥ 2.5-fold the upper limit of normal; estimated glomerular filtration rate (eGFR) < 45 mL/min/1.73m^2^; obesity caused by secondary diseases such as Cushing’s syndrome or pituitary and hypothalamic damage; and use of other medications that may cause weight gain or loss, such as tamoxifen or steroid hormones.

### Procedures and randomization

The study consisted of a screening period, a washout period, a treatment period, and a follow-up period. At screening, patients receiving urate-lowering therapy stopped treatment and underwent a 2-week washout. The patients then underwent a comprehensive baseline examination. Eligible subjects were then randomly assigned to the control and orlistat groups in a 1:1 ratio. For randomization, we used computer- generated numbers distributed in opaque sealed envelopes. Subjects in the control and orlistat groups received 120 mg of placebo and orlistat three times a day within 1 h after meals, respectively. Patients in both groups were instructed to maintain their diet, exercise, and rest habits for nearly 6 months. Both the investigator and the subjects were blinded to subject allocation and the intervention strategies.

### Follow-up

All participants underwent medical history-taking at enrollment, including history of HUA, lifestyle habits (smoking and consumption of alcoholic and sweetened beverages), comorbidities, and family history. Subjects underwent face-to-face visits in the clinic at weeks 0, 4, 8, and 12 during which anthropometric parameters (height, weight, waist circumference, and blood pressure) were measured, biochemical tests for glucose, uric acid, lipids, ALT, AST, γ-glutamate transpeptidase (γ-GT), creatinine, and urea levels were performed, and eGFR was calculated using the CKD-EPI equation. All laboratory measurements were performed using standard methodologies. In addition, at each visit, the occurrence of adverse events including oil spots and gout flares was assessed and noted. If a participant had a gout flare during follow-up, 0.5–1.5 mg colchicine was given over 24 h depending on the severity of the flare, or additional treatment was provided at the discretion of the investigator. At baseline, all patients underwent ultrasound assessments of the knee, ankle, and metatarsophalangeal joints and their associated tendons, and the semi-quantitative OMERACT scoring system was used to assess the double contour (DC) sign, tophus (larger crystal deposits), and aggregations (smaller crystal deposits). At weeks 0 and 12, the controlled attenuation parameters (CAPs) were obtained, and liver stiffness measurements (LSMs) were performed by a trained operator using a FibroScan 502 device (Echosens, Paris, France) to assess changes in liver fat content and the degree of liver fibrosis. The IOI-353 instrument (Yuseong, South Korea) was used to measure changes in body fat composition.

### Statistical analysis

The sample size calculations were based on the success rate of orlistat (65.0%) in terms of reducing liver fat content compared to the control group (17.9%) [13]. A sample of 56 participants (approximately 28 in each group) afforded a power of 95% with a two-sided alpha level < 0.05; even allowing for a 20% dropout rate, the power remained acceptable (> 80%).

The Kolmogorov–Smirnov test was used to check the distributions of parameters. Continuous variables with normal and non-normal distributions are expressed as means ± standard deviations and medians (quartiles), respectively. Categorical variables are expressed as frequencies (percentages). The mean values of normally distributed parameters were compared using the unpaired two-sided Student’s t-test. In other cases, the Mann–Whitney U test was used for group comparisons. Categorical variables were compared using the chi-squared test or the Fisher precision test. Two-tailed *P* values < 0.05 were considered statistically significant. To compare the risk of gout flares between the two groups after initiation of orlistat or placebo treatment, the investigator recorded the number of gout flares per month. For the first gout flare analysis, follow-up ended on the occurrence of the first gout flare or at the end of the study period. A Cox’s proportional hazards model was used to estimate the hazard ratio (HR) of the first gout flare and the 95% confidence interval (CI) during follow-up. The difference between the two groups was compared using the log-rank test. The intention-to-treat population included all participants who had received at least one dose of orlistat or placebo. This population was the primary population for analyses of baseline demographics and clinical characteristics. The per-protocol population included only participants who adhered to the entire study protocol with no deviation; this population served as the population for primary and secondary endpoint analyses. All statistical analyses were performed using SAS 9.4 software (SAS Institute, USA).

## Results

### Baseline characteristics

**Fig. 1:**
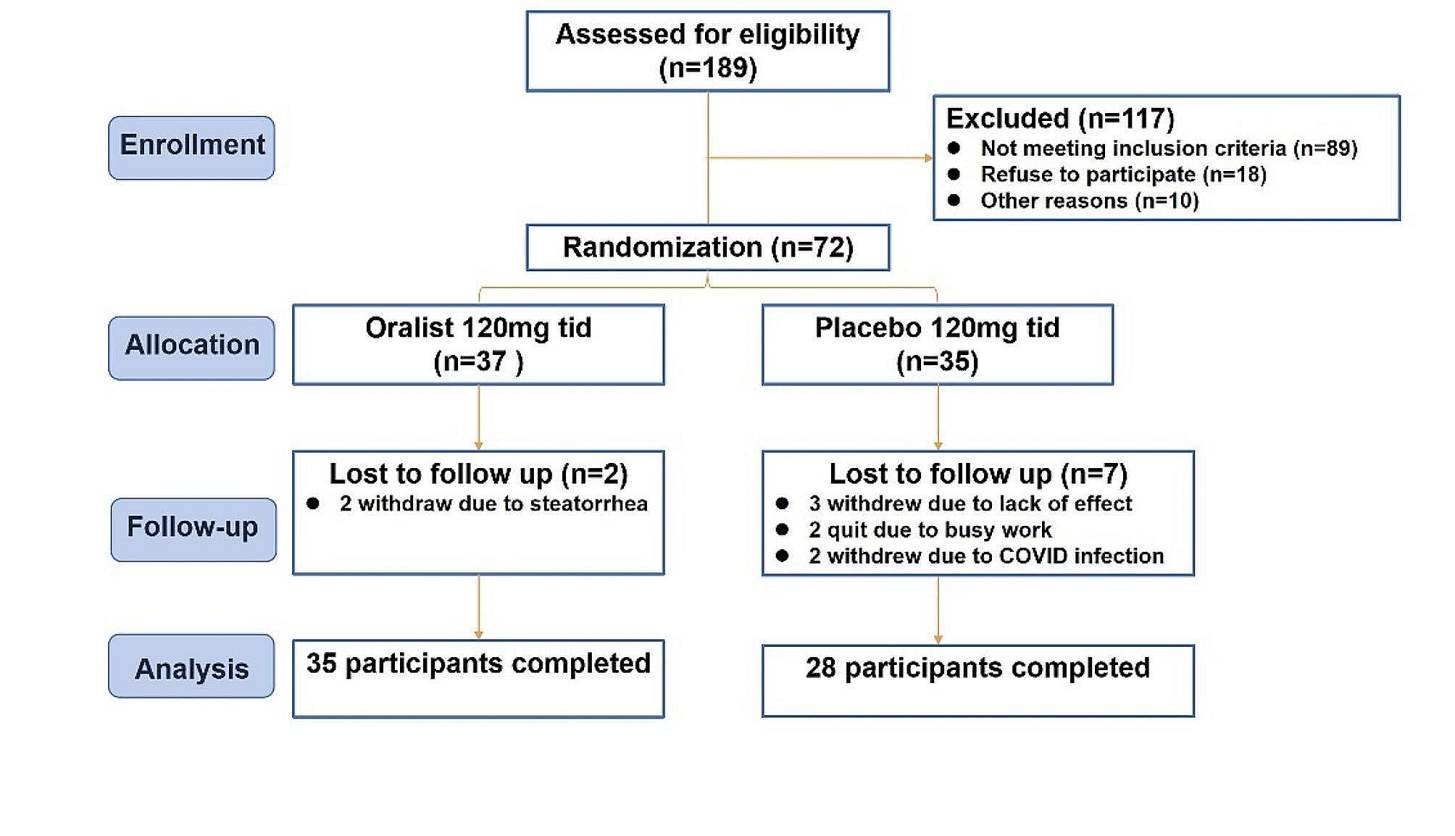
Flow diagram of participants with overweight/obese and hyperuricemia

Of the 72 enrolled participants with overweight/obesity and HUA, 37 were randomly assigned to the orlistat group and 35 to the placebo group (Fig. 1). Two (5.41%) and seven (20.00%) patients in the orlistat and placebo groups, respectively, withdrew from the study, leaving sixty-three subjects for the per-protocol analysis. The most common reasons for discontinuation of treatment were loss to follow-up and withdrawal of consent.

At baseline, the demographic features, biochemical test results, and FibroScan and joint ultrasound results were balanced between both groups. There was no significant difference between the two groups in terms of a history of HUA or lifestyle habits (Table 1).

**Table 1:** Baseline Demographics and Clinical Characteristics

Characteristics	Total (*n* = 72)	Placebo (*n* = 35)	Orlistat (*n* = 37)	P value
**General data**				
Age at entry study (years)	37.00 (30.00,41.00)	37.00 (33.00,41.00)	37.00 (29.00,41.00)	0.685
BMI (kg/m^2^)	29.36 (28.38,32.39)	29.22 (27.93,31.41)	30.33 (28.77,33.29)	0.366
WC (cm)	102.90 (99.63,108.40)	101.50 (98.80,107.80)	103.60 (100.15,111.40)	0.485
Body fat (kg)	27.95 (24.55,30.80)	26.20 (24.05,30.65)	28.15 (25.65,31.18)	0.419
Body muscle (kg)	59.80 (56.50,66.23)	59.80 (55.90,66.23)	60.10 (56.70,66.98)	0.918
**Blood chemistry parameters**				
TC (mmol/L)	5.29 ± 0.92	5.17 ± 0.87	5.41 ± 0.97	0.289
HDL (mmol/L)	1.02 ± 0.15	0.99 ± 0.15	1.04 ± 0.16	0.137
LDL (mmol/L)	3.48 ± 0.73	3.34 ± 0.68	3.62 ± 0.77	0.106
TG (mmol/L)	2.49 ± 1.09	2.47 ± 0.94	2.52 ± 1.23	0.844
Glucose (mmol/L)	5.09 ± 0.49	5.09 ± 0.49	5.09 ± 0.50	0.964
HbA1c (%)	5.50 (5.20,5.80)	5.40 (5.18,5.80)	5.50 (5.30,5.90)	0.732
FINS (uU/ml)	19.96 (14.81,31.00)	19.50 (13.18,31.00)	21.68 (14.98,33.81)	0.520
Fasting C peptide (ng/ml)	3.51 (2.68,4.93)	3.42 (2.64,4.71)	3.55 (2.71,5.03)	0.441
HOMA-IR	3.15 (1.64,6.37)	2.86 (1.66,6.19)	3.78 (1.64,6.40)	0.606
ALT (U/L)	55.11 ± 33.04	52.82 ± 35.89	57.33 ± 30.35	0.569
AST (U/L)	25.20 (19.30,36.20)	20.50 (18.28,34.55)	29.60 (21.65,37.20)	0.534
e-GFR (ml/min/1.73m2)	109.85 (93.95,115.13)	105.70 (93.5,115.2)	111.50 (94.05,116.90)	0.451
Creatinine (µmol/L)	77.00 (73.00,88.00)	80.00 (76.00,89.00)	75.50 (71.00,84.75)	0.353
Urea (mmol/L)	4.45 ± 1.08	4.36 ± 1.22	4.54 ± 0.93	0.492
Uric acid (mg/dl)	9.52 ± 1.60	9.51 ± 1.51	9.53 ± 1.81	0.956
**FibroScan**				
E (kPa)	5.40 (4.60,6.90)	5.25 (4.60,6.18)	5.50 (4.65,7.65)	0.352
CAP (dB/m)	367.00 (316.00,385.00)	341.50 (309.00,382.50)	372.00 (334.00,386.50)	0.324
**Joint ultrasound**				
Aggregates	3.67 ± 3.88	3.61 ± 3.90	3.73 ± 3.91	0.905
Double contour sign	1.15 ± 2.67	1.10 ± 2.68	1.20 ± 2.71	0.945
Tophus	1.15 ± 2.99	1.13 ± 3.00	1.17 ± 3.04	0.961
**History of HUA**				
Duration of HUA (years)	6.00 (3.00,10.00)	6.00 (3.00,13.00)	5.50 (2.25,9.75)	0.847
The highest uric acid before (µmol/L)	628.01 ± 93.97	630.03 ± 94.57	626.22 ± 94.74	0.869
Patients with gout	55 (76.39%)	26 (74.29%)	29 (78.38%)	
Duration of gout (years)	5.00 (2.00,13.00)	6.00 (2.00,13.00)	5.00 (2.00,11.00)	0.583
The number of gout flares before treatment 1 year (times)	1.00 (1.00,3.00)	1.00 (1.00,3.00)	1.00 (1.00,3.00)	0.877
Smoke	23 (31.94%)	11 (31.43%)	12 (32.43%)	0.855
Alcohol	32 (44.44%)	15 (42.86%)	17 (45.95%)	0.977
Sweet drinks				
Occasionally	31 (43.06%)	15 (42.86%)	16 (43.24%)	0.972
Yes	15 (20.83%)	8 (22.86%)	7 (18.92%)	0.815

BMI, body mass index; WC, waist circumference; TC, total cholesterol; HDL, high- density lipoprotein cholesterol; LDL, low-density lipoprotein cholesterol; TG, triglyceride; HbA1c, glycosylated hemoglobin A1c; FINS, fasting insulin; HOMA-IR, homeostasis model assessment of insulin resistance; ALT, alanine aminotransferase; AST, aspartate aminotransferase; e-GFR, estimated glomerular filtration rate; HUA, hyperuricemia

The average BMI of patients in the orlistat group was 30.33 kg/m^2^ and that of patients in the placebo group was 29.22 kg/m^2^. The mean baseline SUA level of patients in the orlistat group was 9.53 [mg/dL] versus 9.51 [mg/dL] in the placebo group. The mean duration of HUA was 5.5 years in the orlistat group and 6.0 years in the placebo group. There were 26 (74.29%) and 29 (78.38%) patients with gout in the orlistat and placebo groups, respectively (Table 1).

### Effects on gout flares

The proportions of patients with gout in the two groups were comparable when considering the total population and all patients who completed follow-up, ranging from 74.29 to 78.38%. The average duration of gout ranged from 5.0 to 6.0 years; the average frequency of gout flares in the year prior to enrolment was 1 episode/year; and the mean tophus score was between 1.13 and 1.17 (Tables 1 and 2).

**Table 2:** Secondary efficacy endpoints

Variables	Total (*n* = 63 )	Placebo (*n* = 28)	Orlistat (*n* = 35)	P
**General data**				
Weight (kg)	-1.92 ± 2.59	-0.76 ± 2.37	-2.85 ± 2.37	0.001
BMI (kg/m^2^)	-0.63 ± 0.83	-0.25 ± 0.75	-0.95 ± 0.77	0.001
WC (cm)	-1.98 ± 2.67	-0.75 ± 2.60	-2.97 ± 2.32	0.001
Body fat (kg)	-1.39 ± 2.11	-0.19 ± 1.69	-2.30 ± 1.95	<0.001
Body muscle (kg)	-0.42 ± 0.89	-0.09 ± 0.87	-0.67 ± 0.83	0.021
Patients with gout	49 (77.78%)	21 (75.00%)	27 (77.14%)	0.843
**Blood chemistry parameters**				
Glucose (mmol/L)	-0.06 ± 0.50	-0.03 ± 0.48	-0.07 ± 0.53	0.776
HbA1c (%)	0.00 (-0.20,0.10)	0.00 (-0.10,0.19)	-0.10 (-0.30,0.10)	0.259
HDL (mmol/L)	-0.05 ± 0.13	0.01 ± 0.13	-0.09 ± 0.10	0.002
LDL (mmol/L)	-0.24 (-0.61,0.21)	0.18 (-0.40,0.44)	-0.55 (-0.67, -0.14)	<0.001
TC (mmol/L)	-0.39 ± 0.75	-0.10 ± 0.64	-0.61 ± 0.76	0.012
TG (mmol/L)	-0.17 (-0.82,0.26)	-0.31 (-0.81,0.13)	-0.14 (-1.11,0.56)	0.268
ALT (U/L)	-4.50 (-21.50,5.00)	-0.85 (-8.33,6.78)	-12.00 (-23.85,2.85)	0.083
AST (U/L)	-1.20 (-7.30,3.20)	0.20 (-2.35,4.20)	-2.80 (-7.85,1.35)	0.172
Urea (mmol/L)	0.20 (-0.40,0.80)	0.30 (-0.43,0.83)	0.10 (-0.35,0.75)	0.609
Creatinine (µmol/L)	0.03 ± 7.38	0.05 ± 7.02	-0.01 ± 7.75	0.983
e-GFR (ml/min/1.73m^2^)	0.38 ± 9.08	5.03 ± 22.52	0.50 ± 10.44	0.390
FINS (uU/ml)	-3.01 (-7.02,0.72)	-2.34 (-7.89,0.99)	-3.08 (-7.24,1.14)	0.856
Fasting C peptide (ng/ml)	-0.59 (-1.35, -0.10)	-0.60 (-1.00, -0.090)	-0.55 (-1.79, -0.07)	0.980
**FibroScan**				
E (kPa)	-0.57 ± 1.34	-0.12 ± 0.90	-0.88 ± 1.52	0.029
CAP (dB/m)	-21.29 ± 32.66	-7.86 ± 36.82	-30.39 ± 26.42	0.013

**Fig. 2:**
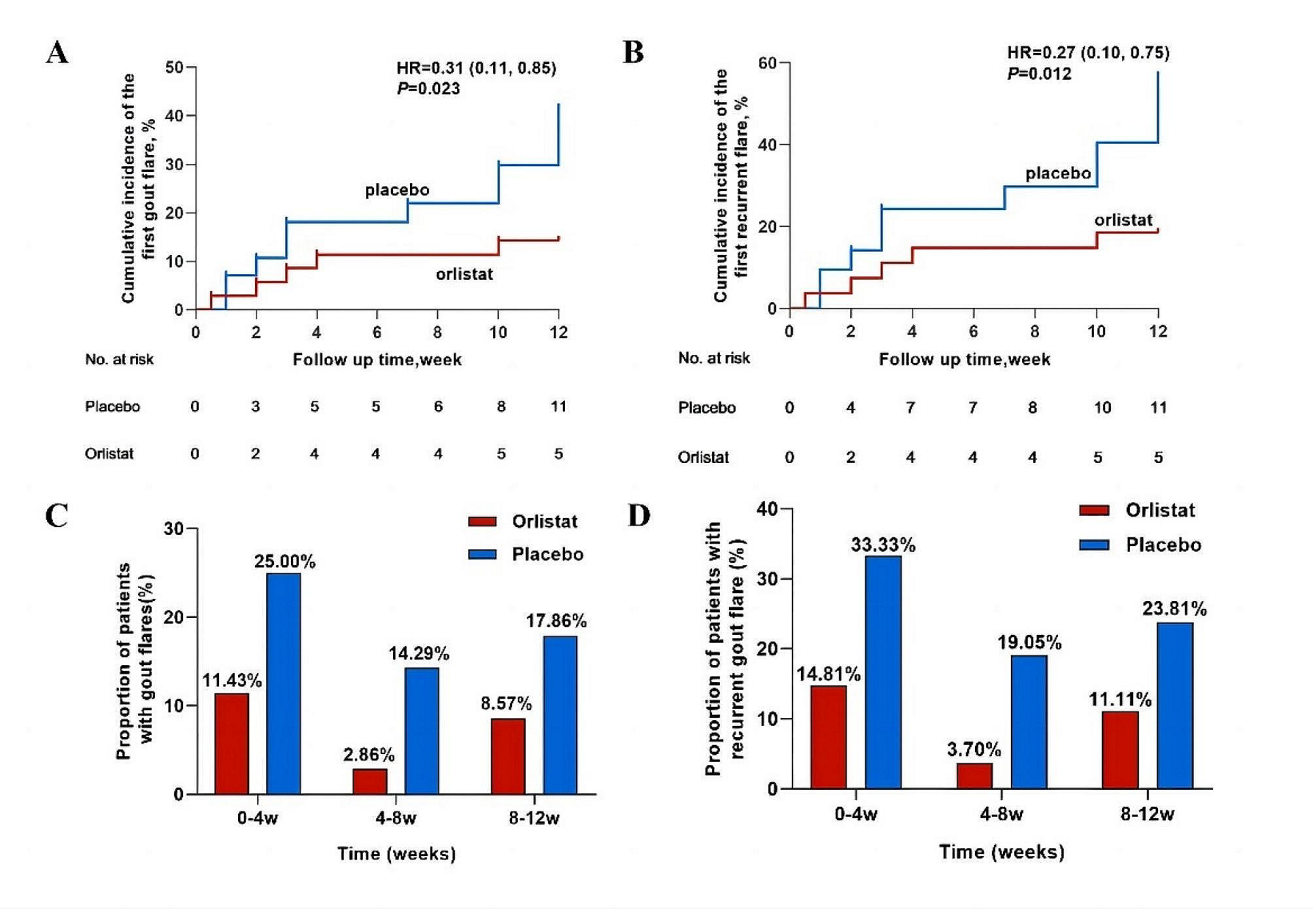
Percentage of patients who experienced gout flares in both groups. **(A)** Cumulative incidence of the first gout flare after initiation of treatment with orlistat or placebo in total participants. **(B)** Cumulative incidence of the first recurrent gout flare after initiation of treatment with orlistat or placebo in participants with gout. **(C)** Proportion of patients with gout flares per month among all participants. **(D)** Proportion of patients with recurrent gout flare per month in participants with gout

During the study period, orlistat treatment was associated with a lower percentage of patients with gout flares (log-rank *P* = 0.023, HR = 0.31, 95% CI 0.11–0.85; Fig. 2a) among all participants and a lower percentage of patients with recurrent gout flares among participants with gout (log-rank *P* = 0.012, HR = 0.27, 95% CI 0.10–0.75; Fig. 2b). All gout flares that were reported were recorded by the investigator. The gout flares were mostly mild or moderate in terms of severity. A total of 11 (39.29%) control group subjects had gout flares compared to 5 (14.28%) in the orlistat group. The proportion of patients with gout flares per month in the total population over time is shown in Fig. 2c. All gout flares occurred in patients with gout, and the proportions of patients with recurrent gout flares per month are shown in Fig. 2d.

### Changes in SUA level

**Fig. 3:**
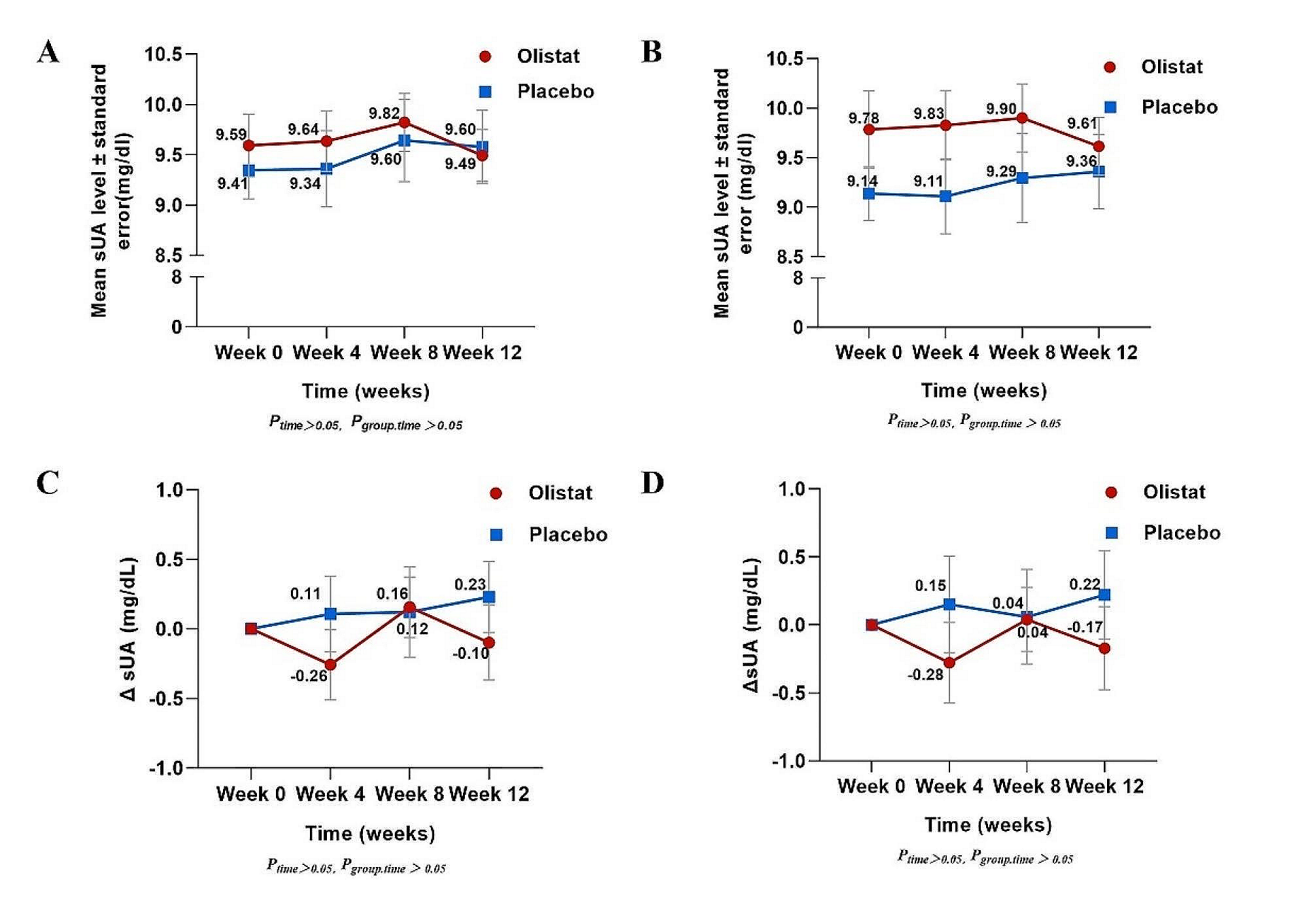
Change in serum uric acid from baseline to week 12. **(A)** Serum uric acid level among all participants. **(B)** Serum uric acid level among participants with gout. **(C)** Change in serum uric acid level among all participants. **(D)** Change in serum uric acid level among participants with gout

During the study period, there was no significant change in the SUA level in either the orlistat group or the placebo group. There was no difference in the SUA change patterns between the two groups (*P*_time_ > 0.05, *P*_group*time_ > 0.05) when considering both the total participants (Fig. 3a) and participants with gout (Fig. 3b). Among all participants, the changes in SUA levels in the orlistat group at 4, 8, and 12 weeks after treatment were − 0.26, 0.16, and − 0.10 mg/dL, respectively. The respective placebo group values were 0.11, 0.12, and 0.23 mg/dL. There was no significant difference in the change in SUA level between the two groups (Fig. 3c). In participants with gout, there was also no significant difference in the changes in SUA level between the two groups (Fig. 3d).

### Changes in obesity and metabolic markers

**Fig. 4:**
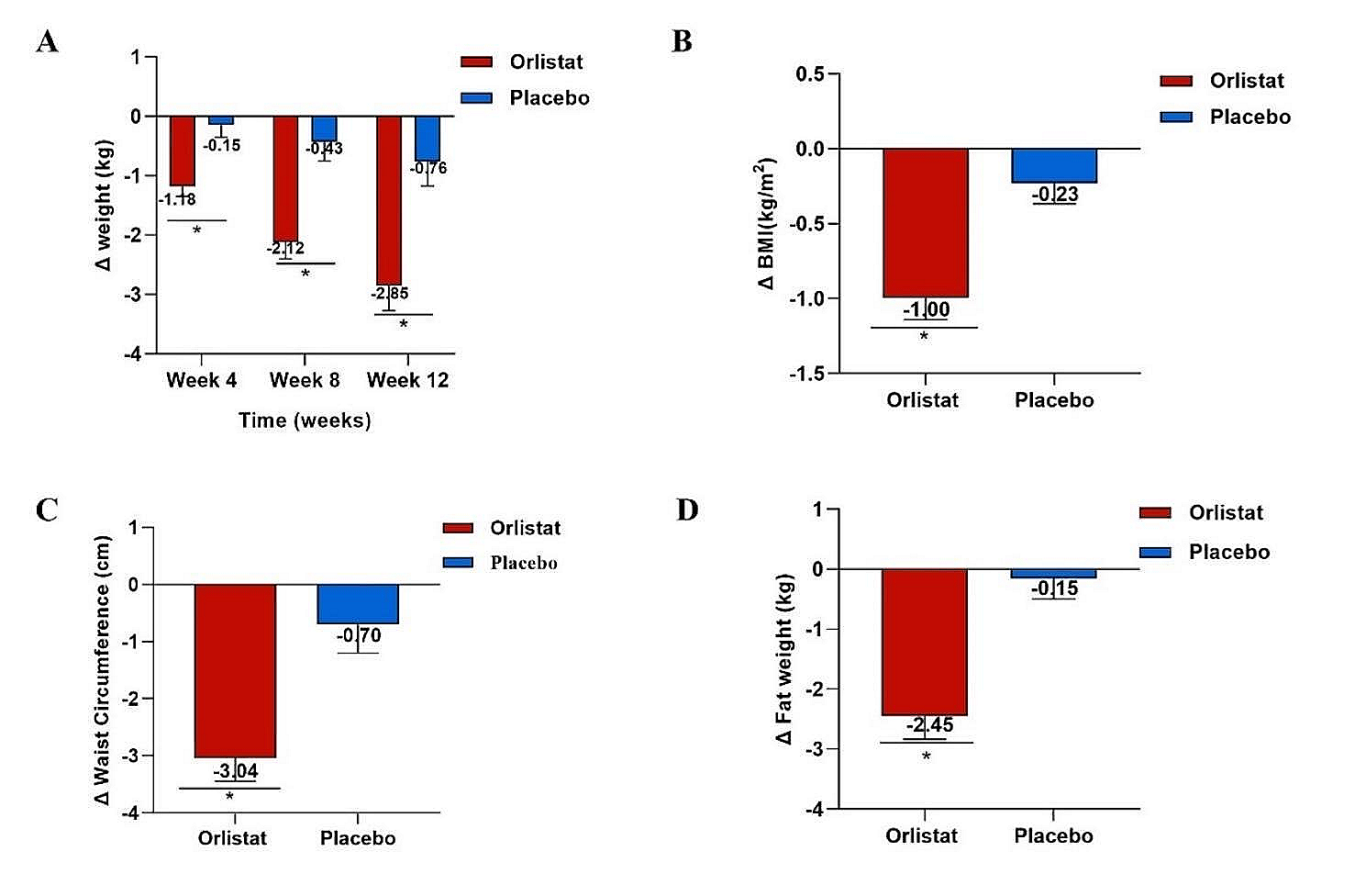
Change in anthropometric parameter measurements over time from baseline to week 12. **(A)** Change in body weight over time from baseline to week 12. **(B)** Change in BMI from baseline to week 12. **(C)** Change in waist circumference from baseline to week 12. **(D)** Change in body fat weight from baseline to week 12. *P<0.05

During the follow-up period, the anthropometric indicators exhibited a downward trend in the orlistat group (Table 2; Fig. 4). Among these measures, patients in the orlistat group showed greater improvements in body weight, BMI, waist circumference, and body fat content at week 12 compared to those in the placebo group (Fig. 4). In addition, the orlistat group also exhibited significant improvements in lipid metabolism, liver fat content, and liver fibrosis. The absolute changes in biological measures, including liver function, kidney function, and glucose metabolism, did not differ over time between the two groups (Table 2).

### Safety

Adverse events and the mental and physical condition of all subjects were recorded by the investigator at each visit. In the orlistat group, 10 (27.03%) participants experienced oil spots, 4 (10.81%) experienced mild diarrhea, and 2 (5.41%) experienced tolerable loss of appetite and abdominal distension. Two (5.41%) participants dropped out because of the negative impact of steatorrhea on work and life. No other adverse events were reported in the placebo group, except gout flares (Table S1).

## Discussion

In this randomized, double-blind, placebo-controlled study, orlistat reduced the rate of gout recurrence in patients with gout. Orlistat was associated with a lower likelihood of gout flares (log-rank *P* = 0.023, HR = 0.31, 95% CI 0.11–0.85) when considering all participants. In contrast, significant lowering of the SUA level was not seen in either group. As expected, the orlistat group performed better than the placebo group in terms of improvements in anthropometric indicators such as body weight, waist circumference, body fat content, lipid metabolism, liver fat content, and liver fibrosis.

In this study, patients with overweight/obesity and HUA treated with orlistat lost an average of 2.85 kg of body weight over a 3-month period, which was significantly higher than that of the control group. This is consistent with previous findings [12, 13].

Obesity has been shown to be associated with more gout flares [18], and weight loss can reduce the frequency of gout flares [19]. For example, gout patients who lost 7.7 kg of weight via diet management experienced a reduced frequency of gout flares, from 2.1 to 0.6 per month [20]. Gout patients who underwent bariatric surgery also exhibited significantly lower rates of gout flare 1 year after surgery [21]. In this study, the proportion of patients with gout was similar between the two groups, and the duration of gout, the frequency of gout flares in the year prior to enrolment, and the joint ultrasound signs were all comparable at baseline. However, during the 12-week follow-up, the rate of gout recurrence was significantly lower in the orlistat group than in the control group. This may be because the weight loss induced by orlistat reduces the frequency of gout flares. In addition, there was a significant decrease in the low-density lipoprotein (LDL) level in the orlistat group, which may also explain the lower rate of gout flares. Previous studies have shown that high LDL levels are associated with frequent gout flares [22].

In addition, gout flares are often induced in obese patients in the early stages of weight loss [21, 23]. Recurrent gout flares often cause patients to lose confidence in the current treatment, making it difficult for them to adhere to the treatment; thus, they are not conducive to long-term weight loss. However, this phenomenon was not noted in the present study. This may be because orlistat downregulates the inflammatory response, in turn suppressing gout flares. No study to date has directly explored the effect of orlistat on gout flares. However, orlistat has been shown to reduce the inflammatory response. In obese rats, orlistat not only increased the levels of antioxidant enzymes but also reduced lipid peroxidation levels, thus alleviating oxidative stress. Moreover, orlistat inhibited the action of nuclear factor kappa-B, which mediates inflammation, and thus improved endothelial dysfunction [24]. In rats with PCOS, orlistat restored the disturbed metabolism of linoleic acid, arachidonic acid, galactose and glycerol and thus attenuated chronic inflammation [25]. Orlistat inhibited the progression of myocardial damage in obese rats by attenuating oxidative stress and inhibiting both the NF-κβ pathway and caspase-dependent apoptosis [26]. Moreover, in obese mice with severe acute pancreatitis, orlistat alleviated adipose tissue necrosis by inhibiting the NLRP3-caspase 1 inflammasome pathway of adipose tissue macrophages [27]. Activation of the NLRP3 inflammasome also plays a crucial role in the acute symptoms of gout, leading to the release of IL-1β and other pro-inflammatory cytokines [28]. Therefore, orlistat is likely to reduce the inflammatory response in obese gout patients, thereby suppressing gout flares. The inhibition of gout flares by orlistat during weight loss could be a useful adjunct treatment for obese gout patients; it would be valuable to reduce gout flares induced in the early stages of weight loss.

Weight loss is thought to be directly related to urate-lowering events. Therapeutic lifestyle changes and bariatric surgery have both been shown to significantly lower SUA levels [29]. However, the effect of orlistat on SUA levels has been an unresolved and controversial issue in patients with HUA. The results show that patients treated with orlistat exhibited an average reduction in SUA levels of 0.10 mg/dL after losing 2.85 kg of weight after 3 months, which is consistent with the dose–response relationship of changes in SUA level with body weight reported in other studies [10]. This study demonstrates, for the first time, that orlistat exhibits no significant urate-lowering effect in patients with overweight/obesity and HUA. This may be attributable to the limited weight loss induced by orlistat, which is not sufficient to lower the SUA level. Orlistat inhibits only about 30% of dietary fat absorption. In 1 year, only 2–5 kg of weight can be lost [30], which is far less than required when the goal for overweight/obese subjects is to achieve a healthy weight.

### Strengths and limitations

This prospective study had several strengths. First, it showed for the first time that orlistat has no direct effect on SUA levels in overweight/obese patients with HUA. Second, the study provided evidence that orlistat might be a useful adjunct therapy during the early stages of weight loss. However, it also had several limitations. First, the study period was not long enough to capture the effects of orlistat on weight loss, lowering of uric acid levels, or gout flares; a longer study period is needed. Second, this study included only male patients with HUA and were thus unable to compare the effects of orlistat on male and female patients and therefore to check for gender differences.

## Conclusions

In summary, this randomized, double-blind, placebo-controlled study demonstrated for the first time that orlistat had no significant effect on SUA levels in patients with overweight/obesity and HUA. In addition, this study found that orlistat was associated with a lower rate of recurrent gout flare during weight loss, suggesting that orlistat may serve as a valuable adjunct therapy in the early stage of weight loss. Further studies are needed to clarify the mechanism by which orlistat prevents gout flares.

### Electronic supplementary material

Below is the link to the electronic supplementary material.

Supplementary Material 1

Supplementary Material 2

Supplementary Material 3

## Data Availability

No datasets were generated or analysed during the current study.
