# Supplementary material for: Efficacy and safety of orlistat in male patients with overweight/obesity and hyperuricemia: results of a randomized, double-blind, placebo-controlled trial
Source: Lipids Health Dis. 2024 Mar 11;23:77. doi: 10.1186/s12944-024-02047-7 (PMC10926609; doi:10.1186/s12944-024-02047-7)
Supplement: Supplementary file 1 — Supplementary Material 1 [file 12944_2024_2047_MOESM1_ESM.docx]

**Table S1. Summary of TEAEs experienced by HUA patients included in two groups.**

| **Event** | **Placebo**  **(n=35)** | **Orlistat**  **(n=37)** |
| --- | --- | --- |
| **Any TEAEs leading to study drug discontinuation** | 0 (0) | 2 (5.41%) |
| Steatorrhea | 0 (0) | 2 (5.41%) |
| Gout flares | 0 (0) | 0 (0) |
| **Drug-related gastrointestinal adverse effects** |  |  |
| Nausea | 0 (0) | 1 (2.70%) |
| Vomiting | 0 (0) | 0 (0) |
|  |  |  |
| Oil spots | 0 (0) | 10 (27.03%) |
| Diarrhea | 0 (0) | 4 (10.81%) |
| Constipation | 0 (0) | 0 (0) |
| Loss of appetite | 0 (0) | 2 (5.41%) |
| Abdominal distension | 0 (0) | 2 (5.41%) |
| Abdominal pain | 0 (0) | 0 (0) |
| Hypoglycemia | 0 (0) | 0 (0) |
| Dizziness | 0 (0) | 0 (0) |
| Upper respiratory tract infection | 0 (0) | 0 (0) |
| Headache | 0 (0) | 0 (0) |
| Gout flares | 11 (39.29%) | 5 (13.51%) |
| Any serious TEAEs | 0 (0) | 0 (0) |
| Death | 0 (0) | 0 (0) |

^^[[1]](#footnote-1)^^ TEAEs, treatment-emergent adverse events.

1. [↑](#footnote-ref-1)
