# Supplementary material for: Efficacy and safety of orlistat in male patients with overweight/obesity and hyperuricemia: results of a randomized, double-blind, placebo-controlled trial
Source: Lipids Health Dis. 2024 Mar 11;23:77. doi: 10.1186/s12944-024-02047-7 (PMC10926609; doi:10.1186/s12944-024-02047-7)
Supplement: Supplementary file 2 — Supplementary Material 2 [file 12944_2024_2047_MOESM2_ESM.pdf]

Textcheck

Textcheck Certificate

Refnum:	24021205
Title:	Efficacy and Safety of Orlistat in Male Patients with Overweight/Obesity and Hyperuricemia: Results of a Randomized, Double-blind, Placebo-controlled Trial
Date:	2024/02/13

We hereby certify that Textcheck has checked and corrected the English in the manuscript named above.

A specialist editor with suitable professional knowledge (M.Sc. or Ph.D./M.D.) reviewed and corrected the English. An English language specialist subsequently checked the paper again. The first language of both editors is English.

Please direct any questions regarding this certificate or the English in the certified paper to: certified@textcheck.com
(Please quote our reference number: '24021205')
