# Supplementary material for: Efficacy and safety of orlistat in male patients with overweight/obesity and hyperuricemia: results of a randomized, double-blind, placebo-controlled trial
Source: Lipids Health Dis. 2024 Mar 11;23:77. doi: 10.1186/s12944-024-02047-7 (PMC10926609; doi:10.1186/s12944-024-02047-7)
Supplement: Supplementary file 3 — Supplementary Material 3 [file 12944_2024_2047_MOESM3_ESM.pdf]

Efficacy and Safety of Orlistat in Male Patient...

By: Haibing Chen

As of: Feb 11, 2024 5:17:42 AM
5,331 words - 21 matches - 18 sources

Similarity Index

9%

Mode: Similarity Report

paper text:

Efficacy and Safety of Orlistat **in** Male **Patients with** Overweight/Obese and **Hyperuricemia** : Results of **a Randomized, Double-Blind, Placebo-Controlled Trial** 18

Shuang Liu1, Xiaojing Lin1, Minghao Tao1, Qi Chen1, Hang Sun1, Yali Han2, Shaoling Yang1, Yining Gao3, Shen Qu1,2*, and Haibing Chen1* Affiliations: 1Department

of Endocrinology and **Metabolism, Shanghai Tenth People's Hospital, Tongji University School of Medicine, 301 Middle Yanchang Road, Shanghai 200072, China** ; 2Shanghai **Center** for Thyroid Disease, **Shanghai Tenth People's Hospital** 6

,

Shanghai 200072, China 3Department **of Endocrinology and Metabolism, Shanghai Sixth People's Hospital, Shanghai Jiao Tong University School of Medicine, 227 Chongqing South Road, Shanghai 200025, China** 1

. Correspondence: Shen Qu, E-mail: qushencn@hotmail.com. ORCiD ID: 0000-0003-0811-7070. Haibing Chen, E-mail: hbchen@tongji.edu.cn. ORCiD ID: 0000-0002-2259-5522. ABSTRACT Background: Obesity is associated with elevated serum uric acid (SUA) and frequent gout flares. Losing weight can lower SUA and reduce gout flares. The effect of orlistat on SUA and gout flares in patients with overweight/obese and hyperuricemia (HUA) has not been extensively studied. This study investigated the effects of orlistat on SUA and gout flares compared to placebo in overweight or obese patients with HUA. Methods: A total of 72 Chinese patients with overweight/obese and HUA were randomly divided into placebo group (35, 48.6%) and orlistat group (37, 51.4%) for 12 weeks. The primary endpoints were relative changes in body weight and SUA, as well as gout flares in the per-protocol population. Results: Orlistat induced a lower proportion of patients with gout flares (log-rank P = 0.023, hazard rate = 0.31, 95% CI 0.11-0.85). There was no significant difference in SUA level between the two groups. The average weight loss of orlistat group was 2.85kg, and the average weight loss in the placebo group was 0.76 kg. The weight loss

effect of the orlistat **group was significantly** better **than that of the control group (P < 0.05).** 13
Conclusions : This study is **the**

first to demonstrate that orlistat has no significant effect on SUA levels in patients with overweight/obese and HUA. The value of orlistat as an adjunct therapy to prevent gout flares during weight loss in patients with HUA was emphasized. Trial registration: Clinicaltrials.gov NCT05496075 Keywords: hyperuricemia; orlistat; uric acid; gout flare Introduction Hyperuricemia (HUA)

is a metabolic disease caused by abnormal **purine metabolism and** /or **reduced excretion of** 15
uric acid in **the** body. The prevalence **of**

HUA in China is 13.3% at present, and shows a trend of younger people. With the development of society and change of lifestyle, the prevalence of HUA will further increase(1). Elevated serum uric acid (SUA) may be deposited in articular and non-articular structures to form monosodium uric acid crystals, leading to gout flares(2), manifested by sudden onset of joint pain, erythema, fever, swelling, and dysfunction(3). In addition, HUA is closely associated with metabolic syndrome, type 2 diabetes, hypertension, cardiovascular disease, chronic kidney disease, and is an independent risk factor for premature mortality(4-6). HUA is considered to be a multifactorial chronic disease related to genetic, environmental and metabolic factors(7). It is particularly emphasized that excess body weight as a major risk factor for the high prevalence of HUA. Epidemiological studies have shown that for every 4 unit increase in body mass index (BMI), SUA can increase by 250μmol/L, and the risk of HUA increases by 7.49 times(8). Animal study has found that obesity promotes increased uric acid production through the hypoxia mechanism of adipocytes(9). The long-term effects of weight loss can help to lower uric acid and relieve gout flares by reducing obesity(10). Given these findings, weight loss is warranted for patients with overweight/obese and HUA. Orlistat is a potent and irreversible inhibitor of lipase that blocks the absorption of triglycerides from the diet(11). Orlistat has been approved by China National Medical Products Administration for the treatment of obese patients. In clinical trials, orlistat has been shown to be effective in improving obesity and related diseases such as metabolic fatty liver disease(12, 13). However, the effect of orlistat on SUA in obese patients remains controversial. In obese patients with metabolic fatty liver disease, there was no significant difference in the change of SUA between the orlistat group and the control group(13, 14). Orlistat combined with Diane-35 significantly reduced SUA in overweight and obese patients with polycystic ovary syndrome, but there was no significant difference compared with Diane-35 alone(15). Orlistat combined with a low-calorie diet (HCD) significantly improved SUA in patients with type 2 diabetes compared to HCD alone(16). In summary, there is no conclusive evidence for the effect of orlistat on SUA. Studies of orlistat in SUA and gout flares in people with overweight/obese and HUA have not been reported and further research is needed. The primary objective of this study was to prospectively evaluate the exact effect of orlistat on urate-lowering and gout flares in patients with overweight/obesity and HUA to inform clinicians' treatment choices. Patients

and Methods Study design This study as **a randomized, double-blind, placebo-controlled trial.** 16

This study

was designed to compare the efficacy of orlistat and placebo in overweight/obese and HUA populations in China. Both orlistat and placebo were provided by HANGZHOU ZHONGMEI-HUADONG PHARMACEUTICAL CO. LTD. From August 2022 to December 2023, a total of 72 subjects with overweight/obese and HUA from the Endocrinology and Metabolism Department of Shanghai Tenth People's Hospital were recruited, of whom 55 (76.39%) were gout patients. The primary endpoints were relative change in SUA and gout flares from baseline to week 12 in the per-protocol population. Gout flares are defined as patient-reported pain that requires rescue medication based on joint symptoms and the nature of the flare. This was recorded through a patient diary and by the investigator at follow-up. Secondary endpoints included changes in body weight, waist circumference, body fat composition, glucose metabolism, liver enzymes, insulin, C-peptides, lipid biomarkers, liver fat content, and degree of liver fibrosis from baseline to week 12 in the per-protocol population. All study data were systematically collected by the investigator using a custom applet. Each participants signed an informed consent prior to entering the program.

This study conducted in accordance with the Declaration of Helsinki and the **Guidelines for Good** 7
Clinical Practice. It **was approved by the** Clinical Research **Ethics Committee of**

the Tenth People's Hospital Affiliated to Tongji University. The study has been registered in ClinicalTrials.gov with the identification code NCT 05496075. Eligibility Inclusion criteria: 1) Age from 18 to 65 years; 2) Male patients with HUA; 3)

BMI (weight in kilograms divided by the square of the **height in meters) ≥ 25.0 kg/m2. The** 11
definitions **of**

gout and HUA are based on the American College of Rheumatology/European Alliance of Associations for Rheumatology gout classification criteria(17). Exclusion criteria: 1) had gout flares in the last two weeks; 2) Abnormal liver function, ALT or AST ≥ 2.5 times the upper limit of normal; 3) eGFR < 45ml/min/1.73m2; 4) Obesity caused by secondary diseases such as Cushing's syndrome, pituitary and hypothalamic damage; 5) Use of other medications that may cause weight gain and loss, such as tamoxifen and steroid hormones. Procedures and Randomization The study consisted of screening period, washout period, treatment period and follow-up period. At screening, patients receiving urate-lowering therapy stopped treatment and underwent a 2-week washout period. The patients were then given a comprehensive baseline examination.

Eligible subjects were then **randomly assigned to the control** and **orlistat groups** in **a 1:1** 5
ratio. **Randomization** is achieved **by computer generated numbers and** distributed **in opaque sealed envelopes. Subjects in the control**

and orlistat groups received 120 mg of placebo and orlistat three times a day within one hour after meals, respectively. Patients in both groups were instructed to maintain their diet, exercise, and rest habits for nearly six months. The allocation of subjects and their intervention strategies are blinded to both the investigator and the subject. Follow-up All participants had a medical history taken at enrollment, including history of HUA, lifestyle habits (smoking, alcohol and drinking sweetened beverages), comorbidities and family history. Subjects underwent face-to-face visits at clinic at weeks 0, 4, 8, and 12 for anthropometric parameter measurements (height, weight, waist circumference and blood pressure) and biochemical tests including glucose, uric acid, lipid levels, alanine aminotransferase (ALT), aspartate aminotransferase (AST), γ-glutamate transpeptidase (γ-GT), creatinine, urea, and estimated glomerular filtration rate (eGFR). EGFR was calculated using the CKD-EPI equation. All laboratory measurements were performed using standard methodologies. In addition, at each visit, the occurrence of adverse events including oil spots and gout flares was asked and assessed. If the participants had a gout flare during follow-up, 0.5-1.5mg colchicine was given over 24 hours depending on the severity of the flare, or additional treatment is provided at the discretion of the investigator. At baseline, all patients had joint ultrasound assessments of the knee, ankle, metatarsophalangeal joints and their associated tendons, and the semi-quantitative OMERACT scoring system was used to assess double contour signs (DC), tophus (larger crystal deposits) and aggregation (smaller crystal deposits). At weeks 0 and 12, controlled attenuation parameter (CAP) and liver stifness measurement (LSM) were performed by a trained operator using FibroScan 502 (Echosens, Paris, France) to assess changes in liver fat content and degree of liver fibrosis. IOI-353 (Yuseong, South Korea) was used to measure the changes in body fat composition. Statistical analysis The sample size calculations were based on the success rate of orlistat (65.0%) in reducing liver fat content compared to the control group (17.9%) (13). A sample of 56 participants (approximately 28 in each group) provided a power of 95% (with a 2-sided alpha level of <0.05), and allowed up to 20% of the dropout rate to maintain acceptable power (>80%).

The Kolmogorov-Smirnov test was used to check **the distribution of parameters. Continuous variables with normal and non-normal distributions are** expressed as **mean ± standard deviation and median (quartile**), respectively. **Categorical variables** are **expressed as frequency (percentage). The mean of the** normally distributed **parameters** was compared using **the unpaired two-sided Student's t test. In other cases, the Mann- Whitney U test was used for** comparison **between** groups. **Categorical variables** were analysed **using Chi-square tests or Fisher** precision **tests** 1

. Bilateral P values < 0.05 was considered statistically significant. To compare the risk of gout flares between the two groups after initiating orlistat or placebo treatment, the investigator recorded the number of gout flares per month.

For the first gout **flare analysis, follow-up ended at the first of the following events: the occurrence of the first** gout **flare** 9

or the end of the study. A Cox proportional hazard model was used to estimate the hazard ratio (HR) of the first gout flare and its 95%CI during follow-up. The difference between the two groups was compared by log-rank test. The intention-to-treat analysis population includes all participants who have received at least one treatment with orlistat or

placebo as the primary analysis population for baseline demographic and clinical characteristic analysis. The per-protocol population only includes participants who adhere to the study protocol without any primary protocol deviation and serves as the primary and secondary endpoint analysis population. All statistical analyses were performed using SAS 9.4. Results Baseline characteristics Of the 72 enrolled participants with overweight/obese and HUA, 37 were randomly assigned to the orlistat group and 35 to the placebo group (Figure 1). 2 (5.41%) and 7 (20.00%) patients in the orlistat and placebo groups withdraw from the study, respectively, leaving 63 subjects for the per-protocol analysis. The most common reasons for discontinuation of treatment are loss of follow-up and withdrawal of consent. At baseline, the results of demography, biochemical test, FibroScan and joint ultrasound results were balanced in both groups. There was no significant difference between the two groups in history of HUA and lifestyle habits (Table 1). The average BMI of patients in the orlistat group was 30.33 and that of patients in the placebo group was 29.22. The mean baseline SUA of patients in the orlistat group was 9.53 versus 9.51

in the placebo group. The mean duration of HUA **was** 5.5 years **in the** orlistat **group and** 10
6. **0** years **in the placebo group**

. There were 26 (74.29%) and 29 (78.38%) patients with gout in the two groups, respectively (Table 1). Effect on gout flares The proportion of patients with gout in the two groups was comparable in the total population and in patients who completed follow-up, ranging from 74.29% to 78.38%. The average duration of gout ranged from 5.0 to 6.0 years, the average frequency of gout flares in the year prior to enrollment was 1 episode/year, and the mean score of tophus was between 1.13 and 1.17 (Table 1, Table 2). During the study period, orlistat was associated with a lower percentage of patients with gout flares (log-rank P = 0.023, HR = 0.31, 95% CI 0.11-0.85; Figure 2a) in total participants and a lower percentage of patients with recurrent gout flares in participants with gout (log-rank P = 0.012, HR = 0.27, 95% CI 0.10-0.75; Figure 2b). Gout flares were reported and recorded by the investigator. Gout flares are mostly mild or moderate in severity. A total of 11 (39.29%) in the control group had gout flares, compared with 5 (14.28%) in the orlistat group. The proportion of patients with gout flares per month in the total population over time is shown in Figure 2c. All of those gout flares occurred in patients with gout, and the proportion of patients with recurrent gout flares per month in the participants with gout is shown in Figure 2d. Changes in SUA level During the study period, there was no significant change in SUA in either the orlistat group or the placebo group. There was no difference in the pattern of SUA change between the two groups (P time >0.05, P group*time >0.05) whether in total participants (Figure 3a) or in participants with gout (Figure 3b). Among all participants, the changes in SUA levels in the orlistat group at 4, 8, and 12 weeks after treatment were -0.26, 0.16, and -0.10mg/dl, respectively. The placebo group was 0.11, 0.12, and 0.23mg/dl, respectively. There was no significant difference in changes in SUA levels between the two groups (Figure 3c). In participants with gout, there was also no significant difference in changes in SUA levels between the two groups (Figure 3d). Changes in obesity and metabolic markers During the follow-up period, anthropometric indicators showed a downward trend in orlistat group (Tables 2 and Figure 4). Among these measures, patients in the orlistat group showed greater improvement in body weight, BMI, waist circumference, and body fat content at week 12 compared with those in the placebo group (Figure 4). In addition, the orlistat group also showed significant improvements in lipid metabolism, liver fat content and liver fibrosis. Absolute changes in biological measures, including liver function, kidney function and glucose metabolism, did not differ over time (Table 2). Safety Adverse events and the patient's mental and physical

condition were recorded by investigator at each visit. In the orlistat group, 10 (27.03%) participants experienced oil spots, 4 (10.81%) participants experienced mild diarrhea, and 2 (5.41%) participants experienced tolerable loss of appetite and abdominal distension. 2 (5.41%) participants dropped out due to the negative impact of steatorrhea on work and life. No other adverse events were reported in the placebo group except for gout flares (Table S1). Discussion This randomized, double-blind, placebo-controlled study shows that orlistat reduces the rate of gout recurrence in patients with gout. Orlistat was associated with a lower percentage of patients with gout flares (log-rank P = 0.023, HR = 0.31, 95% CI 0.11-0.85) in total participants. In contrast, neither group showed a significant advantage in lowering SUA. As expected, Orlistat group was better than placebo group in improving anthropometric indicators such as body weight, waist circumference, body fat content, lipid metabolism, liver fat content and liver fibrosis. In the study, patients with overweight/obese and HUA treated with orlistat lost an average of 2.85kg of body weight over a 3-month period, which was significantly higher than that of the control group. This is consistent with previous findings (12, 13). Obesity has been shown to be associated with increased flares of gout(18) , while weight loss can reduce the frequency of gout flares(19). For example, gout patients who lost 7.7 kg of weight through diet management reduced the frequency of gout flares from 2.1 to 0.6 per month(20). Gout patients who underwent bariatric surgery also had significantly lower rates of gout flares one year after surgery(21). In this study, the proportion of patients with gout was similar between the two groups, and the duration of gout, the frequency of gout flares in the year prior to enrollment, and the joint ultrasound signs were all comparable at baseline. However, during the 12-week follow-up, the rate of gout recurrence was significantly lower in the orlistat group than in the control group. This may be because the weight-loss effect of orlistat reduces the frequency of gout flares. In addition, there was a significant decrease in LDL in the orlistat group, which may also account for the lower rate of gout flares. Because previous studies have shown that high LDL is associated with frequent flares of gout(22). In addition, gout flares are often induced in obese patients in the early stage of weight loss (21, 23). Recurrent gout flares often cause patients to lose confidence in the current treatment, make it difficult to adhere to the treatment, and are not conducive to long-term weight loss. But this phenomenon was not seen in the study. This may be due to orlistat downregulating inflammatory response in the body, which suppresses gout flares. There are currently no known direct studies on the effect of orlistat on gout flares. But orlistat has been shown to reduce the body's inflammatory response. In obese rats, orlistat not only has a favorable effect on antioxidant enzymes, but also reduces lipid peroxidation levels, thus alleviating oxidative stress. Moreover, it can inhibit nuclear factor kappa-B, which mediates inflammation, and improve endothelial dysfunction(24). In Polycystic ovarian syndrome (PCOS) rats, orlistat can restore

the disturbed metabolism of linoleic acid, arachidonic acid, galactose and glycerol, **and** thus attenuate **the chronic inflammation in PCOS rats** 14

(25). Orlistat has been shown to inhibit

the progression of myocardial damage in obese rats by attenuating **oxidative stress, inhibiting** the **NF-κβ pathway, and caspase-dependent apoptosis** 3

(26). Moreover, in obese mice with severe acute pancreatitis, orlistat alleviates adipose tissue necrosis by inhibiting the NLRP3- caspase1 inflammasome pathway of adipose tissue macrophages(27). Activation of the NLRP3 inflammasome also plays a crucial role in the acute symptoms of gout, which leading to the release of IL-1β and other pro-inflammatory cytokines(28). Therefore, Orlistat is likely to reduce the inflammatory response in obese gout patients, thereby suppressing gout flares. The inhibition of gout flares by orlistat during weight loss could be an adjunctive treatment option for obese gout patients to help reduce gout flares induced in the early stages of weight loss. Weight loss is thought to be directly related to urate-lowering. Therapeutic lifestyle changes and bariatric surgery have both been shown to significantly lower SUA(29). But the effect of orlistat on SUA has been an unresolved and controversial issue in patients with HUA. The results showed that patients treated with orlistat had an average reduction in SUA levels of 0.10 mg/dl after losing 2.85 kg of weight at 3 months, which is consistent with the dose-response relationship of changes in SUA levels with body weight in other studies(10). The study demonstrates for the first time that orlistat has no significant urate- lowering effect in patients with overweight/obese and HUA. This may be due to the limited weight loss of orlistat, which is not enough to lower SUA. Orlistat inhibits only about 30% of dietary fat absorption. In 1 year, only 2 - 5 kg of weight can be lost(30), which is far from the goal of achieving a healthy weight for large weight people. Addressed The study has several advantages. First, this study prospectively identified for the first time that orlistat has no direct effect on SUA in overweight/obese patients with HUA. Second, this study provide evidence for the potential of orlistat as an adjuvant therapy for the early stages of weight loss. There are several limitations to the study. First, the study period was not long enough to capture the effects of orlistat on weight loss, uric acid- lowering, and gout flares over a longer study period. Second, this study included only male patients with HUA and were unable to compare the combined effect of orlistat between male and female patients while excluding gender differences in the results. Conclusions In summary, this randomized, double-blind, placebo-controlled study demonstrated for the first time that orlistat has no significant effect on SUA levels in patients with overweight/obese and HUA. In addition, this study found that orlistat was associated with a lower rate of recurrent gout flare during weight loss, suggesting that orlistat may be used as an adjunctive therapy in the early stage of weight loss. Further studies are needed to clarify the mechanism of orlistat on preventing gout flare.

Data availability statement All data generated or analysed during this study are available from the corresponding authors on **reasonable request**. Declarations Competing interests **The authors declare** that they have **no conflict of interest** 2

.

Funding This research was funded by the Key Technologies Research and Development Program [grant number: 2019YFA0904500], **National** 4

Key Research and Development Program of China [2022YFC2503300], the National Natural Science Foundation of China [grant numbers: 81870606,82170904], the Shanghai Municipal Health Commission Clinical Research Project [202240130], and the Program for Research-oriented Physician of Shanghai Tenth People's Hospital. Author's

contribution Conceptualization, S.L.; Formal analysis, S.L. and Y.H.; Funding acquisition, H.C.; Investigation, S.L., X.L., H.S., S.Y. and Y.G.; Methodology, S.L. and Q.C.; Project administration, H.C.; Software, S.L. and Y.H.; Supervision, S.Q. and H.C.; Visualization, S.L.; Writing – original draft, S.L.; Writing – review & editing, S.L., M.T. and H.C. All authors reviewed the manuscript.

Acknowledgements We thank the trial staff as well as all people who agreed to participate in this study 1

. Ethics

approval This study was approved by the Ethics Committee of Shanghai Tenth People's Hospital. It was conducted in accordance with the Declaration of Helsinki and the Guidelines for Good Clinical Practice. Informed Consent All participants gave written informed consent before taking part in the study. References 1

1. Liu R, Han C, Wu D, Xia X, Gu J, Guan H, et al. Prevalence of Hyperuricemia and Gout in Mainland China from 2000 to 2014: A Systematic Review and Meta-Analysis. Biomed Res Int. 2015;2015:762820. 2. Dalbeth N, Gosling AL, Gaffo A, Abhishek A. Gout. Lancet. 2021;397(10287):1843-55. 3. Klück V, Jansen T, Janssen M, Comarniceanu A, Efdé M, Tengesdal IW, et al. Dapansutrile, an oral selective NLRP3 inflammasome inhibitor, for treatment of gout flares: an open-label, dose-adaptive, proof-of-concept, phase 2a trial. Lancet Rheumatol. 2020;2(5):e270-e80. 4. Si K, Wei C, Xu L, Zhou Y, Lv W, Dong B, et al. Hyperuricemia and the Risk of Heart Failure: Pathophysiology and Therapeutic Implications. Front Endocrinol (Lausanne). 2021;12:770815. 5. Yokose C, McCormick N, Choi HK. The role of diet in hyperuricemia and gout. Curr Opin Rheumatol. 2021;33(2):135-44. 6. Lee SJ, Oh BK, Sung KC. Uric acid and cardiometabolic diseases. Clin Hypertens. 2020;26:13. 7. Nakayama A, Matsuo H, Nakaoka H, Nakamura T, Nakashima H, Takada Y, et al. Common dysfunctional variants of ABCG2 have stronger impact on hyperuricemia progression than typical environmental risk factors. Sci Rep. 2014;4:5227. 8. Palmer TM, Nordestgaard BG, Benn M, Tybjærg-Hansen A, Davey Smith G, Lawlor DA, et al. Association of plasma uric acid with ischaemic heart disease and blood pressure: mendelian randomisation analysis of two large cohorts. Bmj. 2013;347:f4262. 9. Tsushima Y, Nishizawa H, Tochino Y, Nakatsuji H, Sekimoto R, Nagao H, et al. Uric acid secretion from adipose tissue and its increase in obesity. J Biol Chem. 2013;288(38):27138-49. 10. Zhu Y, Zhang Y, Choi HK. The serum urate-lowering impact of weight loss among men with a high cardiovascular risk profile: the Multiple Risk Factor Intervention Trial. Rheumatology (Oxford). 2010;49(12):2391-9. 11. Schcolnik-Cabrera A, Chávez-Blanco A, Domínguez-Gómez G, Taja-Chayeb L, Morales-Barcenas R, Trejo-Becerril C, et al. Orlistat as a FASN inhibitor and multitargeted agent for cancer therapy. Expert Opin Investig Drugs. 2018;27(5):475-89. 12. Valladales-Restrepo LF, Sánchez-Ramírez N, Usma-Valencia AF, Gaviria-Mendoza A, Machado-Duque ME, Machado-Alba JE. Effectiveness, persistence of use, and safety of orlistat and liraglutide in a group of patients with obesity. Expert Opin Pharmacother. 2023;24(4):535-43. 13. Feng X, Lin Y, Zhuo S, Dong Z, Shao C, Ye J, et al. Treatment of obesity and metabolic-associated fatty liver disease with a diet or orlistat: A randomized controlled trial. The American Journal of Clinical Nutrition. 2023;117(4):691-700. 14. Ye J, Wu Y, Li F, Wu T, Shao C, Lin Y, et al. Effect of orlistat on liver fat content in patients with

nonalcoholic fatty liver disease with obesity: assessment using magnetic resonance imaging-derived proton density fat fraction. Therap Adv Gastroenterol. 2019;12:1756284819879047. 15. Song J, Ruan X, Gu M, Wang L, Wang H, Mueck AO. Effect of orlistat or metformin in overweight and obese polycystic ovary syndrome patients with insulin resistance. Gynecol Endocrinol. 2018;34(5):413-7. 16. Didangelos TP, Thanopoulou AK, Bousboulas SH, Sambanis CL, Athyros VG, Spanou EA, et al. The ORLIstat and CArdiovascular risk profile in patients with metabolic syndrome and type 2 DIAbetes (ORLICARDIA) Study. Curr Med Res Opin. 2004;20(9):1393-401. 17. Neogi T, Jansen TL, Dalbeth N, Fransen J, Schumacher HR, Berendsen D, et al. 2015 Gout classification criteria: an American College of Rheumatology/European League Against Rheumatism collaborative initiative. Ann Rheum Dis. 2015;74(10):1789-98. 18. Bajpai R, Muller S, Mallen C, Watson L, Richette P, Hider SL, et al. Onset of comorbidities and flare patterns within pre-existing morbidity clusters in people with gout: 5-year primary care cohort study. Rheumatology (Oxford). 2021;61(1):407-12. 19. Danve A, Sehra ST, Neogi T. Role of diet in hyperuricemia and gout. Best Pract Res Clin Rheumatol. 2021;35(4):101723. 20. Dessein PH, Shipton EA, Stanwix AE, Joffe BI, Ramokgadi J. Beneficial effects of weight loss associated with moderate calorie/carbohydrate restriction, and increased proportional intake of protein and unsaturated fat on serum urate and lipoprotein levels in gout: a pilot study. Ann Rheum Dis. 2000;59(7):539-43. 21. Romero-Talamás H, Daigle CR, Aminian A, Corcelles R, Brethauer SA, Schauer PR. The effect of bariatric surgery on gout: a comparative study. Surg Obes Relat Dis. 2014;10(6):1161-5. 22. Uhlig T, Karoliussen LF, Sexton J, Kvien TK, Haavardsholm EA, Hammer HB. Lifestyle factors predict gout outcomes: Results from the NOR-Gout longitudinal 2-year treat-to-target study. RMD Open. 2023;9(4). 23. Kang EH, Lee EY, Lee YJ, Song YW, Lee EB. Clinical features and risk factors of postsurgical gout. Ann Rheum Dis. 2008;67(9):1271-5. 24. Hamza RZ, Alsolami K. Ameliorative effects of Orlistat and metformin either alone or in combination on liver functions, structure, immunoreactivity and antioxidant enzymes in experimentally induced obesity in male rats. Heliyon. 2023;9(8):e18724. 25. Yang J, Wang E, Chen W, Xu B, Chen C, Zhang G, et al. TMT-Based Proteomics Analysis of the Intervention Effect of Orlistat on Polycystic Ovary Syndrome Rats Induced by Letrozole Combined with a High-Fat Diet. ACS Omega. 2023;8(28):24831- 40. 26. Othman ZA, Zakaria Z, Suleiman JB, Mustaffa KMF, Jalil NAC, Wan Ghazali WS, et al. Orlistat Mitigates Oxidative Stress-Linked Myocardial Damage via NF-κβ- and Caspase-Dependent Activities in Obese Rats. Int J Mol Sci. 2022;23(18). 27. Xu T, Sheng L, Guo X, Ding Z. Free Fatty Acid Increases the Expression of NLRP3-Caspase1 in Adipose Tissue Macrophages in Obese Severe Acute Pancreatitis. Dig Dis Sci. 2022;67(6):2220-31. 28. So AK, Martinon F. Inflammation in gout: mechanisms and therapeutic targets. Nat Rev Rheumatol. 2017;13(11):639-47. 29. Chalès G. How should we manage asymptomatic hyperuricemia? Joint Bone Spine. 2019;86(4):437-43. 30. Ballinger A, Peikin SR. Orlistat: its current status as an anti-obesity drug. Eur J Pharmacol. 2002;440(2-3):109-17. Tables Table 1. Baseline Demographics and Clinical Characteristics. Characteristics Total Placebo Orlistat P value (n=72) (n=35) (n=37) General data Age at entry study (years) BMI (kg/m2) WC (cm) Body fat (kg) Body muscle (kg) 37.00 (30.00,41.00) 29.36 (28.38,32.39) 102.90 (99.63,108.40) 27.95 (24.55,30.80) 59.80 (56.50,66.23) 37.00 (33.00,41.00) 29.22 (27.93,31.41) 101.50 (98.80,107.80) 26.20 (24.05,30.65) 59.80 (55.90,66.23) 37.00 (29.00,41.00) 30.33 (28.77,33.29) 103.60 (100.15,111.40) 28.15 (25.65,31.18) 60.10 (56.70,66.98) 0.685 0.366 0.485 0.419 0.918 Blood chemistry parameters

TC (mmol/L) HDL (mmol/L) LDL (mmol/L) TG (mmol/L) Glucose (mmol/L

) HbA1c (%) FINS (uU/ml) Fasting C peptide (ng/ml) HOMA-IR ALT (U/L) AST (U/L) e-GFR (ml/min/1.73m2) 5.29±0.92 1.02±0.15 3.48±0.73 2.49±1.09 5.09±0.49 5.50 (5.20,5.80) 19.96 (14.81,31.00) 3.51 (2.68,4.93) 3.15 (1.64,6.37) 55.11±33.04 25.20 (19.30,36.20) 109.85 (93.95,115.13) 5.17±0.87 5.41±0.97 0.289 0.99±0.15 1.04±0.16 0.137 3.34±0.68 3.62±0.77 0.106 2.47±0.94 2.52±1.23 0.844 5.09±0.49 5.09±0.50 0.964 5.40 (5.18,5.80) 5.50 (5.30,5.90) 0.732 19.50 0.520 (13.18,31.00) 21.68 (14.98,33.81) 3.42 (2.64,4.71) 3.55 (2.71,5.03) 0.441 2.86 (1.66,6.19) 3.78 (1.64,6.40) 0.606 52.82±35.89 57.33±30.35 0.569 20.50 0.534 (18.28,34.55) 29.60 (21.65,37.20) 105.70 (93.5,115.2) 111.50 (94.05,116.90) 0.451 416 417 418 419 420 Creatinine (μmol/L) Urea (mmol/L) Uric acid (mg/dl) FibroScan E (kPa) CAP (dB/m) Joint ultrasound Aggregates Double contour sign Tophus History of HUA Duration of HUA (years) The highest uric acid before (μmol/L) Patients with gout Duration of gout (years) The number of gout flares before treatment 1 year (times) Smoke 77.00 (73.00,88.00) 4.45±1.08 9.52±1.60 5.40 (4.60,6.90) 367.00 (316.00,385.00) 3.67±3.88 1.15±2.67 1.15±2.99 6.00 (3.00,10.00) 628.01±93.97 55 (76.39%) 5.00 (2.00,13.00) 1.00 (1.00,3.00) 23 (31.94%) 80.00 (76.00,89.00) 4.36±1.22 9.51±1.51 5.25 (4.60,6.18) 341.50 (309.00,382.50) 3.61±3.90 1.10±2.68 1.13±3.00 6.00 (3.00,13.00) 630.03±94.57 26 (74.29%) 6.00 (2.00,13.00) 1.00 (1.00,3.00) 11 (31.43%) 75.50 (71.00,84.75) 4.54±0.93 9.53±1.81 5.50 (4.65,7.65) 372.00 (334.00,386.50) 3.73±3.91 1.20±2.71 1.17±3.04 5.50 (2.25,9.75) 626.22±94.74 29 (78.38%) 5.00 (2.00,11.00) 1.00 (1.00,3.00) 12 (32.43%) 0.353 0.492 0.956 0.352 0.324 0.905 0.945 0.961 0.847 0.869 0.583 0.877 0.855 Alcohol Sweet drinks Occasionally Yes 32 (44.44%) 31 (43.06%) 15 (20.83%) 15 (42.86%) 15 (42.86%) 8 (22.86%) 17 (45.95%) 16 (43.24%) 7 (18.92%) 0.977 0.972 0.815 BMI, body mass index; WC, waist circumference; TC, total cholesterol;

HDL, high- density lipoprotein cholesterol; LDL, low-density lipoprotein cholesterol; TG, triglyceride; 8 **HbA1c** , glycosylated **hemoglobin A1c** ; FINS, fasting insulin; **HOMA-IR, homeostasis model** assessment **of insulin resistance** ; ALT, alanine aminotransferase; **AST**

, aspartate aminotransferase; e-GFR, estimated glomerular filtration rate; HUA, hyperuricemia. 421 Table 2. Secondary efficacy endpoints. Variables Total (n=63) General data Weight (kg) -1.92±2.59 BMI (kg/m2) -0.63±0.83 WC (cm) -1.98±2.67 Body fat (kg) -1.39±2.11 Body muscle (kg) -0.42±0.89 Patients with gout 49 (77.78%) Placebo (n=28) -0.76±2.37 -0.25±0.75 -0.75±2.60 -0.19±1.69 -0.09±0.87 21 (75.00%) Orlistat (n=35) -2.85±2.37 -0.95±0.77 -2.97±2.32 -2.30±1.95 -0.67±0.83 27 (77.14%) P 0.001 0.001 0.001 < 0.001 0.021 0.843 Blood parameters chemistry Glucose (mmol/L) -0.06±0.50 -0.03±0.48 -0.07±0.53 0.776 422 423 424 425 426 427 HbA1c (%) HDL (

mmol/L) LDL (**mmol/L**) TC (**mmol/L**) TG (**mmol/L**) ALT (**U/L**) AST (**U/L)** 12 **Urea (mmol/L) Creatinine (μmol/L**

) e-GFR (ml/min/1.73m2) FINS (uU/ml) Fasting C peptide (ng/ml) 0.00 (-0.20,0.10) -0.05±0.13 -0.24 (-0.61,0.21) -0.39±0.75 -0.17 (-0.82,0.26) -4.50 (-21.50,5.00) -1.20 (-7.30,3.20) 0.20 (-0.40,0.80) 0.03±7.38 0.38±9.08 -3.01 (-7.02,0.72) -0.59 (-1.35, -0.10) 0.00 (-0.10,0.19) 0.01±0.13 0.18 (-0.40,0.44) -0.10±0.64 -0.31 (-0.81,0.13) -0.85 (-8.33,6.78) 0.20 (-2.35,4.20) 0.30 (-0.43,0.83) 0.05±7.02 5.03±22.52 -2.34 (-7.89,0.99) -0.60 (-1.00, -0.090) -0.10 (-0.30,0.10) -0.09±0.10 -0.55 (-0.67, -0.14) -0.61±0.76 -0.14 (-1.11,0.56) -12.00 (-23.85,2.85) -2.80 (-7.85,1.35) 0.10 (-0.35,0.75) -0.01±7.75

0.50±10.44 -3.08 (-7.24,1.14) -0.55 (-1.79, -0.07) 0.259 0.002 < 0.001 0.012 0.268 0.083 0.172 0.609 0.983 0.390 0.856 0.980 FibroScan E (kPa) CAP (dB/m) -0.57±1.34 -21.29±32.66 -0.12±0.90 -7.86±36.82 -0.88±1.52 -30.39±26.42 0.029 0.013 Figure legends Figure 1： Flow diagram of participants with overweight/obese and hyperuricemia. Figure 2： Percentage of patients who experienced gout flares in both groups. (A) Cumulative incidence of the first gout flare after initiation of treatment with orlistat or placebo in total participants. (B) Cumulative incidence of the first recurrent gout flare after initiation of treatment with orlistat or placebo in participants with gout. (C) Proportion of patients with gout flares per month among all participants. (D) Proportion of patients with recurrent gout flare per month in participants with gout. Figure 3： Change in serum uric acid from baseline to week 12. (A) Serum uric acid level among all participants. (B) Serum uric acid level among participants with gout. (C) Change in serum uric acid level among all participants. (D) Change in serum uric acid level among participants with gout. Figure 4： Change in anthropometric parameter measurements over time from baseline to week 12. (A) Change in body weight over time from baseline to week 12. (B) Change in BMI from baseline to week 12. (C) Change in waist circumference from baseline to week 12. (D) Change in body fat weight from baseline to week 12. *P < 0.05 442 1 2 3 4 5 6 7 8 9 10 11 12 13 14 15 16 17 18 19 20 21 22 23 24 25 26 27 28 29 30 31 32 33 34 35 36 37 38 39 40 41 42 43 44 45 46 47 48 49 50 51 52 53 54 55 56 57 58 59 60 61 62 63 64 65 66 67 68 69 70 71 72 73 74 75 76 77 78 79 80 81 82 83 84 85 86 87 88 89 90 91 92 93 94 95 96 97 98 99 100 101 102 103 104 105 106 107 108 109 110 111 112 113 114 115 116 117 118 119 120 121 122 123 124 125 126 127 128 129 130 131 132 133 134 135 136 137 138 139 140 141 142 143 144 145 146 147 148 149 150 151 152 153 154 155 156 157 158 159 160 161 162 163 164 165 166 167 168 169 170 171 172 173 174 175 176 177 178 179 180 181 182 183 184 185 186 187 188 189 190 191 192 193 194 195 196 197 198 199 200 201 202 203 204 205 206 207 208 209 210 211 212 213 214 215 216 217 218 219 220 221 222 223 224 225 226 227 228 229 230 231 232 233 234 235 236 237 238 239 240 241 242 243 244 245 246 247 248 249 250 251 252 253 254 255 256 257 258 259 260 261 262 263 264 265 266 267 268 269 270 271 272 273 274 275 276 277 278 279 280 281 282 283 284 285 286 287 288 289 290 291 292 293 294 295 296 297 298 299 300 301 302 303 304 305 306 307 308 309 310 311 312 313 314 315 316 317 318 319 320 321 322 323 324 325 326 327 328 329 330 331 332 333 334 335 336 337 338 339 340 341 342 343 344 345 346 347 348 349 350 351 352 353 354 355 356 357 358 359 360 361 362 363 364 365 366 367 368 369 370 371 372 373 374 375 376 377 378 379 380 381 382 383 384 385 386 387 388 389 390 391 392 393 394 395 396 397 398 399 400 401 402 403 404 405 406 407 408 409 410 411 412 428 429 430 431 432 433 434 435 436 437 438 439 440 441 1 2 3 4 5 6 7 8 9 10 11 12 13 14 15 16 17 18 19 20 21 22 23 24

sources:

1 — 157 words / 3% - Crossref
S. Liu, H. Sun, S. Yang, N. Liang, Y. Gao, S. Qu, H. Chen. "Clustering of gout-related comorbidities and their relationship with gout flares: a data-driven cluster analysis of eight comorbidities", Journal of Endocrinological Investigation, 2023

2 — 25 words / < 1% match - from 27-Jan-2024 12:00AM
WWW.MDPI.COM

3 — 19 words / < 1% match - Internet from 24-Sep-2022 12:00AM
www.mdpi.com

4 15 words / < 1% match - Internet from 14-Dec-2022 12:00AM
www.mdpi.com

5 27 words / < 1% match - Crossref
Xiongcai Feng, Yansong Lin, Shuyu Zhuo, Dong Zhi, Congxiang Shao, Junzhao Ye, Bihui Zhong. "Treatment of Obesity and Metabolic-Associated Fatty Liver Disease with a Diet or Orlistat: A Randomized Controlled Trial", The American Journal of Clinical Nutrition, 2023

6 26 words / < 1% match - Internet from 02-Jan-2022 12:00AM
buscador.una.edu.ni

7 23 words / < 1% match - from 06-Jul-2023 12:00AM
d.docksci.com

8 23 words / < 1% match - from 23-Jan-2024 12:00AM
www.dovepress.com

9 21 words / < 1% match - Crossref
Jie Wei, Hyon K. Choi, Nicola Dalbeth, Xiaoxiao Li, Changjun Li, Chao Zeng, Guanghua Lei, Yuqing Zhang. "Gout Flares and Mortality After Sodium-Glucose Cotransporter-2 Inhibitor Treatment for Gout and Type 2 Diabetes", JAMA Network Open, 2023

10 18 words / < 1% match - Crossref
P.-G. Sator, M. O. Sator, J. B. Schmidt, H. Nahavandi, S. Radakovic, J. C. Huber, H. Hönigsmann. "A prospective, randomized, double-blind, placebo-controlled study on the influence of a hormone replacement therapy on skin aging in postmenopausal women", Climacteric, 2009

11 18 words / < 1% match - Internet from 13-Oct-2020 12:00AM
e-dmj.org

12 18 words / < 1% match - from 27-Dec-2023 12:00AM
healthdocbox.com

13 17 words / < 1% match - from 25-Aug-2023 12:00AM
www.qk.sjtu.edu.cn

14 17 words / < 1% match - from 02-Sep-2023 12:00AM
www.unboundmedicine.com

15 16 words / < 1% match - Internet from 25-Feb-2023 12:00AM
www.frontiersin.org

16 15 words / < 1% match - from 21-Oct-2023 12:00AM
acta.tums.ac.ir

17 15 words / < 1% match - Internet from 05-Oct-2018 12:00AM
bmccomplementalternmed.biomedcentral.com

18

15 words / < 1% match - from 07-Feb-2024 12:00AM
www.springermedizin.de
